# Ectopic Axillary Breast during Systemic Lupus

**DOI:** 10.1155/2012/403932

**Published:** 2012-08-08

**Authors:** Besma Ben Dhaou, Fatma Boussema, Zohra Aydi, Lilia Baili, Lilia Rokbani

**Affiliations:** Internal Medicine Department of Habib, Thameur Hospital, Tunis 1008, Tunisia

## Abstract

Many breast changes may occur in systemic lupus erythematosus. We report a 41-year-old woman with lupus who presented three years after the onset of lupus an ectopic mammary gland confirmed by histological study.

## 1. Introduction

The interruption of the migration of the milk ducts of the axilla to the midclavicular line can occur in a 2% to 6% of female embryos resulting in ectopic breast tissue. This gland undergoes the same physiological and pathological influences as the normal breast tissue. The systemic lupus erythematosus (SLE) seems to influence the mammary gland in fact several cases of mastitis, breast tumor benign or malignant were reported in patients with SLE. In this regard, we report the observation of a patient who developed a supernumerary in the course of SLE.

## 2. Case Report 

**Figure 1:**
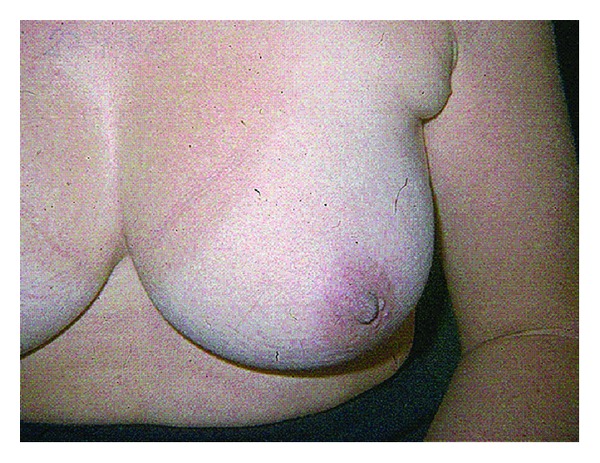
Ectopic axillary breast.

A 41-year-old woman was admitted, in 2005, for exploration of a left axillary mass. She is followed since 2002 for SLE diagnosed at 8 ACR criteria. In 2003, the SLE was complicated by glomerular nephropathy with pure mesangial alterations and diffuse membranous glomerulonephritis so grade II and V of the WHO. The patient was then treated with corticosteroids at a dose of 1 mg/kg/day with usual degression associated with synthetic antimalarial treatment, with a favorable outcome without relapsing for two years. The examination on admission showed a patient in good general condition, cushingoid facies, and left axillary swollen, soft, 2x3 cm without signs of inflammation (Figure 1).

**Figure 2:**
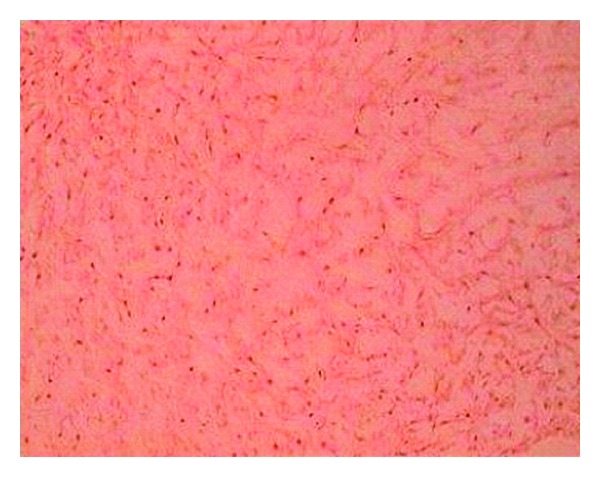
Histology: Breast tissue.

The lymph nodes were free. Breast examinations found no palpable nodule or nipple discharge. The ultrasound exploration of the mass showed an aspect compatible with ectopic mammary gland. The patient received a resection of this mass. Histological examination confirmed the existence of a supernumerary breast (Figure 2). Seven-year followup, so to 2012, shows a stable condition without recurrence of the mass.

## 3. Discussion

The originality of our observation lies in the fact that the ectopic breast was misunderstood and that it has developed after the lupus disease. Several observations have reported hypertrophy of the mammary gland in systemic lupus. It has been reported that majority of the cases of mammary hypertrophy occur during either pregnancy or puberty, supporting the theory of abnormal stimulation of mammary tissue by circulating hormones [1]. Substantial evidence of the immunoregulatory function of estradiol, testosterone, progesterone, DHEA, and pituitary hormones, including PRL, has supported the hypothesis that they modulate the incidence and severity of SLE [2, 3].

Oestrogen stimulates CD8+ and CD4+ Tcells, Bcells, macrophages, the release of certain cytokines, and the expression of both HLA and endothelial cell adhesion molecules. Another potentially important effect of estradiol may be its ability to reduce apoptosis in self-reactive Bcells, thus promoting selective maturation of B-cells with high affinity for anti-DNA [4].

Progesterone and PRL also affect immune activity. Progesterone downregulates T-cell proliferation, while lupus flares have been associated with hyperprolactinaemia [5, 6]. Elevated serum PRL levels have been reported in patients with SLE, and hyperprolactinaemia occurs in approximately 15–31% of patients with SLE, and these elevated PRL levels do appear to correlate with clinical disease activity, as well as antinuclear antibody and anti-dsDNA titreS [7–9]. Chuang and Molitch [10] noted that differences in peripheral PRL or 3 lymphocyte production, rather than pituitary production of PRL, may worsen disease activity and breast tissue hypersensitivity. In our case, very likely ectopic axillary breast was embryologic remnant, but hypertrophy has occurrence after the onset of systemic lupus.

Duffy et al. reported the case of a patient aged 24 who developed breast gigantism in a course of the lupus disease [11]. Similarly another case of refractory gynecomastia to tamoxifen has been reported requiring the use of an iterative surgical resection [12]. Borges da Silva reported the occurrence of a hamartoma in an unknown axillary 4 mammary gland in a patient with systemic lupus [13]. Recent studies have shown an increased risk of breast cancer compared to a female population of same age [14]. Breast changes in systemic lupus are related to estrogen hyperstimulation, hyperprolactinaemia, and elevated IGF1.

## 4. Conclusion

Many breast changes may occur in SLE. The fact remains that before any changes in the mammary gland in a patient with lupus a biopsy must be done and because, as evidenced by the literature review, lupus is a risk factor for benign breast tumors but also and especially malignant tumors.
